# Anti-Müllerian Hormone as a Biomarker for Predicting Testicular Sperm Extraction Outcomes in Azoospermic Patients: A Comprehensive Systematic Review and Meta-Analysis

**DOI:** 10.3390/ijms262311643

**Published:** 2025-12-01

**Authors:** Dragoș Puia, Marius Ivănuță, Mihaela Corlade-Andrei, Ovidiu Daniel Bîcă, Bogdan Doroftei, Cătălin Pricop

**Affiliations:** 1 Grigore T. Popa University of Medicine and Pharmacy Iasi, 700115 Iasi, Romania; drdragos83@yahoo.com (D.P.); corladeandrei.mihaela@yahoo.com (M.C.-A.); bogdandoroftei@gmail.com (B.D.); bobopricop@yahoo.com (C.P.); 2 Department of Urology, “Dr. C.I. Parhon” Clinical Hospital, 700503 Iasi, Romania; 3 Center for Morphological and Spectroscopic Analysis of Urinary Stones” Michel Daudon”, 700503 Iasi, Romania; 4 Emergency Care Department, “Sf. Spiridon” County University Emergency Hospital, 700111 Iasi, Romania; 5 Origyn Fertility Center, Palace Street No. 3C, 700032 Iasi, Romania; 6 Clinical Hospital of Obstetrics and Gynecology “Cuza Voda”, Cuza Voda Street No. 34, 700038 Iasi, Romania

**Keywords:** anti-Müllerian hormone, TESE, sperm retrieval rate, azoospermia, male infertility

## Abstract

Male infertility represents a major clinical and societal issue, with azoospermia being one of its most severe forms. Anti-Müllerian Hormone (AMH) has been proposed as a potential biomarker for predicting testicular sperm extraction (TESE) outcomes in men with non-obstructive azoospermia (NOA). This study aimed to systematically evaluate the association between AMH levels and sperm retrieval success. We included studies on men with NOA reporting TESE outcomes by AMH level, excluding those without full text or with insufficient data. When cohorts overlapped, the most complete study was used, following PICO criteria focused on AMH measurements and sperm retrieval rates (SRR). A comprehensive search identified 133 potentially relevant publications. Of these, 11 studies published between 2006 and 2023, including 1280 patients, met the inclusion criteria. Pooled analyses were performed using random-effects models. This meta-analysis was recorded in the PROSPERO database (registration ID: CRD420251065256). Reported SRRs ranged from 30.35% to 76.27%. Meta-analysis of nine studies assessing serum AMH concentrations revealed significant heterogeneity (I^2^ = 88%). Elevated serum AMH was negatively associated with SRR (standardized mean difference [SMD] = −2.58; 95% CI: −4.73 to −0.44; *p* < 0.00001). In contrast, seminal plasma AMH levels (two studies) showed no significant association with SRR (I^2^ = 82%). Similarly, preoperative FSH levels (nine studies) did not demonstrate a consistent association with SRR, despite higher mean concentrations in patients with successful TESE (*p* = 0.02; SMD = −4.86; 95% CI: −9.07 to −0.66). Serum AMH levels are significantly associated with TESE outcomes in men with NOA. However, the predictive value of AMH and other hormonal markers is limited by high inter-individual variability and overlapping values between successful and unsuccessful cases. These findings underscore the complexity of NOA and highlight the need to interpret hormonal markers within a broader clinical and biochemical context.

## 1. Introduction

Male infertility represents a major clinical and societal challenge, affecting a substantial proportion of couples seeking to conceive. It is estimated that male factors contribute to nearly half of all infertility cases worldwide, with significant emotional, psychological, and financial consequences for affected couples. Among the various etiologies, azoospermia—defined as the complete absence of spermatozoa in the ejaculate—is recognized as one of the most severe forms of male infertility. Epidemiological data indicate that azoospermia is present in approximately 1% of the general male population and accounts for up to 10–15% of cases among infertile men [1]. Importantly, azoospermia is not a uniform condition: it can be broadly classified as obstructive azoospermia (OA), resulting from blockage of the reproductive tract, and non-obstructive azoospermia (NOA), caused by intrinsic testicular failure and impaired spermatogenesis. While OA may be surgically correctable, NOA remains a far more complex and heterogeneous disorder, often with uncertain treatment outcomes.

The clinical evaluation of male infertility typically involves a comprehensive diagnostic approach, including medical history, physical examination, semen analysis, and hormonal profiling. Hormonal markers such as follicle-stimulating hormone (FSH), luteinizing hormone (LH), and anti-Müllerian hormone (AMH) are routinely measured to assess testicular function and to elucidate the underlying pathophysiology [2,3]. Elevated FSH and LH levels are frequently observed in men with NOA, reflecting disruption of the hypothalamic–pituitary–gonadal axis because of impaired spermatogenesis [2]. However, while FSH and LH provide useful indirect information, they lack sufficient accuracy to reliably predict the presence or absence of spermatozoa within the testes. Consequently, there is continued interest in identifying more specific, non-invasive biomarkers that could refine clinical decision-making.

The development of assisted reproductive technologies, particularly intracytoplasmic sperm injection (ICSI), has transformed the treatment landscape of male infertility. Men with NOA now have the possibility of achieving biological fatherhood, provided that viable spermatozoa can be retrieved directly from testicular tissue [4,5]. The surgical techniques most frequently used include conventional testicular sperm extraction (TESE) and microdissection TESE (micro-TESE). Despite technical advances, sperm retrieval rates (SRR) remain highly variable, ranging widely across studies and clinical settings. Multiple factors—including patient age, testicular volume, histopathological subtype, and hormonal milieu—have been investigated as potential predictors of success [6]. Given the invasive nature of TESE, along with its associated physical and psychological burden, there is a critical need for reliable, non-invasive biomarkers that can predict the likelihood of successful sperm retrieval in men with NOA [6,7].

In this context, attention has increasingly focused on biomarkers reflecting Sertoli cell function and spermatogenic potential. Among these, AMH has emerged as a particularly promising candidate [4,6]. AMH is a glycoprotein belonging to the transforming growth factor-β (TGF-β) family, secreted by Sertoli cells during foetal development and persisting, at lower levels, throughout adult life. In males, AMH plays a fundamental role in sexual differentiation by inducing regression of the Müllerian ducts, thereby preventing the development of female internal reproductive structures. Beyond its developmental function, AMH continues to serve as a marker of Sertoli cell activity and, indirectly, of spermatogenic status [4]. Because Sertoli cells provide structural and nutritional support to developing germ cells, alterations in AMH secretion may reflect the functional integrity of the seminiferous epithelium, which is often impaired in NOA [6].

Several observational studies have suggested that serum and seminal plasma AMH concentrations differ significantly across fertility categories. Fertile men typically present with the highest AMH levels, whereas men with NOA exhibit the lowest concentrations. Moreover, AMH has been positively correlated with sperm count and sperm quality, supporting its potential role as a biomarker of spermatogenesis [8]. In azoospermic men undergoing TESE, both serum and seminal AMH have been investigated as predictors of sperm retrieval, with conflicting results. Some studies report that lower AMH levels are associated with a reduced likelihood of sperm retrieval, while others find no significant association. These discrepancies may reflect differences in study design, patient populations, assay methodologies, or cutoff values.

Despite these uncertainties, AMH remains an attractive biomarker because it integrates both developmental and functional aspects of Sertoli cell physiology. Unlike FSH and LH, which primarily reflect hypothalamic–pituitary feedback, AMH may provide more direct information on the intratesticular environment. Its potential utility as a predictor of TESE outcomes lies in its ability to capture subtle differences in Sertoli cell function and spermatogenic activity that are not discernible through routine hormonal assays.

However, the clinical application of AMH in this context is currently limited by several factors. First, published studies are heterogeneous, with relatively small sample sizes and variable designs. Second, there is no universally accepted threshold for AMH levels in predicting TESE success, leading to inconsistent findings. Third, AMH is often assessed in isolation, whereas male infertility is multifactorial, and a single biomarker may not provide sufficient predictive accuracy. These limitations underscore the need for a comprehensive synthesis of the available evidence.

Current major guidelines (e.g., American Urological Association, European Association of Urology) do not routinely recommend AMH for male infertility/TESE prediction, underscoring the knowledge gap your review aims to fill. Therefore, the present systematic review and meta-analysis were undertaken to evaluate the association between AMH levels critically measured in both serum and seminal plasma, and sperm retrieval outcomes in men with NOA undergoing TESE. By pooling data from published studies, we aimed to clarify whether AMH represents a reliable biomarker for predicting TESE success and to identify the limitations and gaps that should guide future research.

## 2. Materials and Methods

This systematic review and meta-analysis was conducted in accordance with the Preferred Reporting Items for Systematic Reviews and Meta-Analyses (PRISMA) guidelines [9]. The research question was formulated using the Population, Intervention, Comparison, and Outcome (PICO) framework. The study population consisted of males with non-obstructive azoospermia (NOA). The intervention of interest was testicular sperm extraction (TESE), and the comparison was the analysis of AMH levels between patients with successful and failed TESE. The primary outcome was the sperm retrieval rate (SRR), while secondary outcomes included associations between AMH and reproductive hormone parameters.

### 2.1. Data Sources and Search Strategy

Systematic literature searches were performed in MEDLINE, Scopus and Embase using a predefined strategy. The search terms combined free-text and controlled vocabulary, as follows:

*‘AMH’ [MeSH Terms] AND (‘non-obstructive azoospermia’ [All Fields] OR ‘NOA’ [All Fields]) AND (‘testicular sperm extraction’ [All Fields]) AND (‘sperm parameters’ [MeSH Terms] OR ‘TESE’ [All Fields])*

The search covered all available records up to 31 December 2023. Reference lists of relevant reviews and included articles were also screened manually to identify additional eligible studies.

### 2.2. Eligibility Criteria

We included studies enrolling men with NOA that reported TESE outcomes stratified by AMH levels. Articles were excluded if the full text was unavailable or if they were case reports, conference abstracts, letters, or comments providing insufficient data for analysis. In cases of overlapping cohorts, the most recent or comprehensive study was retained. The PICO criteria used are as follows: Population: adult male patients with azoospermia; Intervention: serum and seminal plasma AMH levels, in addition to serum and seminal AMH concentrations, we also analyzed those of FSH, Inhibin B, and total testosterone; Comparator: high vs. low AMH groups or correlation with continuous AMH; and the Outcome: sperm retrieval rate.

### 2.3. Study Selection and Data Extraction

Two reviewers independently screened all titles and abstracts for eligibility. Full texts of potentially relevant articles were then assessed in detail. Discrepancies were resolved by consensus, and if necessary, adjudicated by a third reviewer.

A standardized data extraction form was used to collect study-level information, including design, population characteristics, type of biological sample (serum or seminal plasma), AMH measurement units, reported outcomes, and risk-of-bias assessments. 

### 2.4. Statistical Analysis

AMH concentrations were reported in different units across studies. To harmonize results, we converted values expressed in ng/dL into pmol/L using the JAMA 2001 Systeme International conversion factors for laboratory components [10].

Heterogeneity across studies was quantified using the I^2^ statistic, with values >50% indicating significant heterogeneity. Pooled effect sizes were calculated using a random-effects model to account for clinical and methodological variability. We reported standardized mean differences (SMDs), odds ratios (ORs), and 95% confidence intervals (CIs). Analyses were performed using Review Manager (RevMan), version 5.4 (Copenhagen:The Cochrane Collaboration, 2020).

### 2.5. Quality Assessment

Considering that all included studies were observational, the Newcastle–Ottawa Scale (NOS) was applied to assess the risk of bias. This tool evaluates studies across three domains: selection, comparability, and outcome assessment.

### 2.6. Registration

This meta-analysis was prospectively registered in the PROSPERO international database (registration ID: CRD420251065256).

## 3. Results

### 3.1. Study Selection

**Figure 1:**
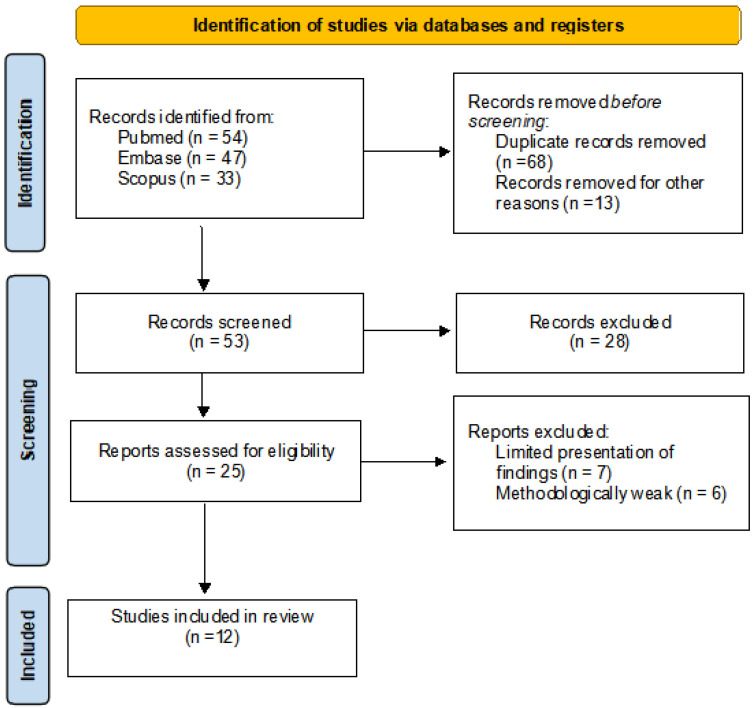
PRISMA (Preferred Reporting Items for Systematic Reviews and Meta-Analyses) 2020 flow diagram.

Following the systematic search, a total of 133 potential publications were identified. After removal of duplicates and detailed screening of titles, abstracts, and full texts, 11 studies met the inclusion criteria and were included in the final analysis. In Figure 1, the PRISMA 2020 flow diagram illustrates the study selection process, from initial database search to final inclusion.

### 3.2. Study Characteristics

The included studies were published between 2006 and 2023, enrolling a total of 1306 patients with NOA. Reported sperm retrieval rates (SRR) ranged from 30.35% to 76.27%, reflecting variability across cohorts in sample size, TESE technique, and patient characteristics. Details of study features, including design, sperm retrieval method, SRR, and Newcastle–Ottawa Scale (NOS) scores, are summarized in Table 1.

### 3.3. Serum AMH and Sperm Retrieval Outcomes

**Figure 2:**
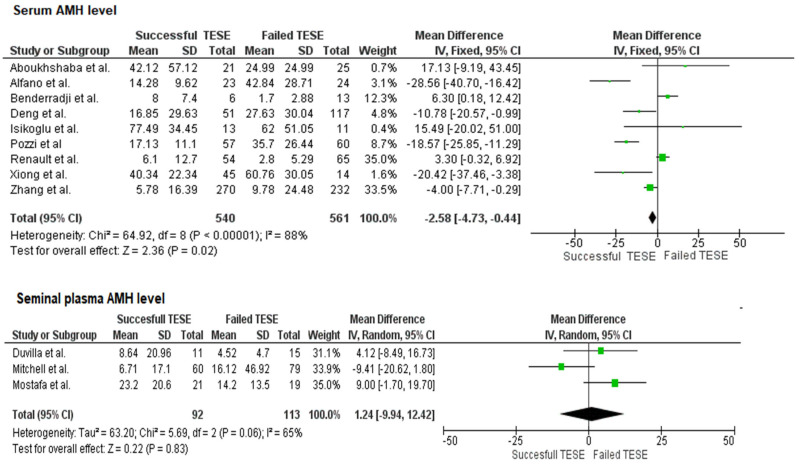
Forest plots for serum and seminal plasma AMH levels.

Significant methodological heterogeneity existed across studies. Seminal plasma studies predominantly used ELISA methodology and reported results in pmol/L, while serum studies reported in ng/mL. Nine studies reported on serum AMH concentrations. The pooled analysis demonstrated high heterogeneity (I^2^ = 88%). Using a random-effects model, elevated serum AMH levels were found to be negatively associated with SRR (SMD = −2.58; 95% CI: −4.73 to −0.44; *p* < 0.00001). The corresponding forest plots for both serum and seminal plasma AMH levels are shown in Figure 2.

### 3.4. Seminal AMH and Sperm Retrieval Outcomes

Three studies (n = 207) assessed seminal plasma AMH levels. Despite moderate heterogeneity (I^2^ = 65%), the random-effects model showed no significant association between seminal AMH concentrations and SRR.

### 3.5. FSH Levels and TESE Outcomes

**Figure 3:**
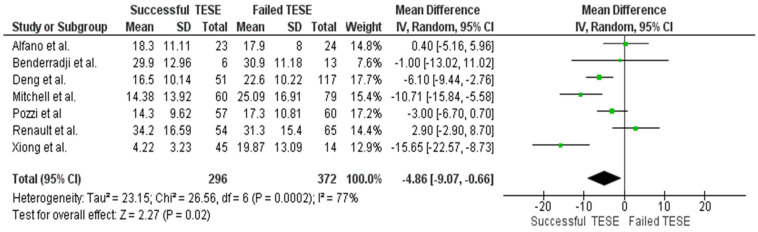
Forest plot of FSH levels.

Nine of the included studies (n = 668) reported preoperative serum FSH levels. Patients with successful TESE had higher mean FSH concentrations; however, the pooled analysis did not confirm a statistically robust association with SRR (SMD = −4.86; 95% CI: −9.07 to −0.66; *p* = 0.02). The forest plot for FSH levels is presented in Figure 3.

### 3.6. Inhibin and Total Testosterone Levels

**Figure 4:**
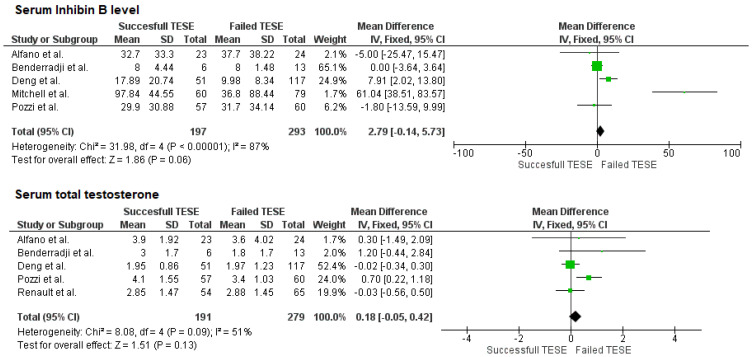
Forest plots for serum Inhibin B and total testosterone levels.

Of the included studies, five were able to extract data on serum Inhibin B and total testosterone concentrations. As shown in Figure 4, neither preoperative concentrations of Inhibin B nor total testosterone were significantly correlated with the outcome of the intervention, *p* = 0.06, respectively *p* = 0.13.

### 3.7. Combined Biomarker Models

We also analysed the combination of different markers. The findings were very heterogeneous. Only four studies evaluated this aspect. The results are shown in Table 2.

### 3.8. Quality Assessment

Study quality, as assessed by the Newcastle–Ottawa Scale (NOS), ranged from 4 to 8, with a mean score of 6.63, indicating an overall moderate methodological quality. Lower scores were primarily attributable to small sample sizes and short follow-up periods.

## 4. Discussion

Hormonal biomarkers are fundamental tools in the assessment and management of male infertility, offering valuable information on testicular function, spermatogenesis, and the underlying causes of impaired fertility. Among these, AMH has attracted considerable attention due to its unique biological properties and clinical relevance. AMH reflects the functional status of Sertoli cells, which are central to supporting germ cell development and maintaining the seminiferous epithelium [21]. In adult men, circulating AMH provides insight into Sertoli cell activity, which is particularly relevant in the context of NOA, a condition characterized by severely impaired or absent spermatogenesis [22]. Indeed, Turhan et al. reported that AMH levels vary according to aetiology, with distinctive patterns in patients with non-obstructive azoospermia [23]. Research has shown that serum and seminal plasma AMH levels range markedly among various male fertility classifications, with the lowest amounts seen in men with NOA and the highest in fertile men. The strong correlation between AMH levels and sperm concentration further underscores the significance of AMH in evaluating spermatogenic state. Furthermore, seminal AMH levels are markedly elevated in fertile men relative to those with oligoasthenoteratozoospermia, and are generally undetectable in OA patients, highlighting its diagnostic significance [5,16].

The rationale for evaluating AMH as a predictor of TESE outcomes is grounded in its biological role and its potential to serve as a non-invasive biomarker of testicular function. Benderradji et al. demonstrated that low serum AMH concentrations may indicate Sertoli and germ cell dysfunction, particularly in specific subgroups such as men with non-mosaic Klinefelter syndrome, where AMH below a threshold value was associated with 100% sensitivity for negative sperm retrieval [1]. This suggests that AMH could be used to identify patients with minimal likelihood of sperm retrieval and spare them unnecessary invasive procedures.

A distinction must be made between serum and seminal plasma AMH. While our findings showed a significant association for serum AMH, seminal plasma AMH failed to demonstrate predictive value. This may be explained by proteolytic activity in seminal fluid, which could degrade AMH and impair measurement accuracy [11]. Plotton et al. further observed that plasma AMH differs significantly between obstructive and non-obstructive azoospermia, supporting its diagnostic role in distinguishing etiologies, even though its direct correlation with TESE success remains inconsistent [24]. Other factors, such as vitamin D status, may also influence AMH expression, complicating its interpretation [3].

The predictive capacity of AMH has been debated, with both positive and negative associations reported. Mendelson et al. concluded that AMH, FSH, and inhibin B may serve as optional markers for sperm retrieval, but none alone reliably predicts the presence of mature spermatozoa [25]. While AMH has shown good diagnostic accuracy for differentiating normozoospermia and oligozoospermia (AUC = 0.857, sensitivity 70%, specificity 96% at ≤2.5 ng/mL) [21], its direct application to TESE outcomes remains less certain. Age and clinical history further affect AMH values, since both AMH and inhibin B decline with age, necessitating age-adjusted reference ranges.

In clinical conditions like NOA, characterized by spermatogenic failure, inhibin B levels are typically decreased, suggesting compromised Sertoli cell function or a reduced number of germ cells. The authors indicate that inhibin B is likely the physiologically relevant form of inhibin in men, highlighting its significance in male reproductive endocrinology. The clinical utility of inhibin B as a biomarker has been investigated for its role in predicting the presence of sperm in testicular tissue, especially in men undergoing TESE for azoospermia. Numerous studies indicate that decreased inhibin B levels correlate with a diminished probability of successful sperm retrieval in TESE procedures [24,25,26]. The predictive accuracy of inhibin B is constrained due to significant overlap in its concentrations among men with both successful and unsuccessful sperm retrieval outcomes. This limitation indicates that although inhibin B offers important insights into Sertoli cell function and spermatogenic status, it must be considered in conjunction with other clinical and hormonal factors to improve its predictive utility.

Inhibin B levels are affected by multiple factors, such as age, testicular volume, and the specific cause of azoospermia. In cases of primary testicular failure, inhibin B levels are typically low, while in secondary hypogonadism, these levels may be preserved or only slightly reduced. The variability requires a detailed interpretation of inhibin B measurements in clinical practice. 

The relationship between AMH and inhibin B has also been studied, with significant correlations reported, but without consistent predictive value for TESE success [15]. Testicular histology remains the most reliable predictor of sperm retrieval, albeit requiring invasive biopsy [26,27]. Schwarzkopf et al. emphasized that Leydig cell function may, in some contexts, be more predictive than Sertoli cell markers, particularly in patients with Klinefelter syndrome [28]. While AMH and inhibin B may be more sensitive indicators of spermatogenic activity than FSH, their predictive value in TESE remains under debate [8,27].

In specific histological patterns such as Sertoli cell-only syndrome (SCOS), AMH levels are often markedly reduced, reflecting the absence or immaturity of Sertoli cells. Conversely, higher AMH concentrations in some adults may indicate the persistence of immature Sertoli cells, usually associated with impaired spermatogenesis and reduced probability of sperm retrieval [29]. Schwarzkopf et al. noted that although significant differences in AMH exist between NOA patients and fertile controls, most values still fall within the normal range, which limits its ability to serve as a universal biomarker [28].

In the context of azoospermia, specifically NOA, FSH levels are commonly employed as a biomarker to assess the functional condition of the seminiferous epithelium and the probability of successful sperm retrieval during TESE procedures. Increased FSH levels are frequently noted in males with compromised spermatogenesis, indicating a compensatory reaction to decreased inhibin B and altered Sertoli cell activity. This hormonal feedback loop is characteristic of testicular failure, as the pituitary gland elevates FSH output to stimulate the impaired testicular tissue. However, FSH, traditionally considered a key marker, remains controversial. Some studies report associations between lower FSH and higher SRR [6,30], whereas others and several meta-analyses failed to confirm its predictive accuracy [6,12]. This inconsistency reflects the heterogeneity of testicular pathology in NOA, where elevated FSH may occur in many conditions, not all of which exclude focal spermatogenesis. The predictive accuracy of FSH for TESE outcomes is not definitive. Schwarzkopf et al. assert that although increased FSH signifies spermatogenic failure, there exists substantial overlap in FSH levels among individuals with both successful and failed sperm retrieval [28]. This restriction occurs because FSH indicates overall testicular activity rather than specific regions of spermatogenesis, which may continue even in testes with generally suboptimal performance [5]. Thus, certain individuals with excessive FSH levels may still possess localized areas of active spermatogenesis, facilitating effective sperm retrieval, whereas others with only modestly raised FSH may not.

The type of surgical technique must also be considered. Micro-TESE generally yields higher SRRs than conventional TESE, with reported rates between 61–73% and, in selected histologies, as high as 96.8% [31,32]. In contrast, conventional TESE typically achieves SRRs of 40–50% [33]. Notwithstanding its therapeutic significance, TESE has many dangers and restrictions that must be taken into account while advising patients and formulating care plans. A key worry is the inconsistent success rate of sperm retrieval, affected by underlying testicular disease, hormonal environment, and individual patient features [34]. The surgical procedure of TESE carries inherent risks, such as testicular damage, hematoma formation, infection, and the potential compromise of residual testicular function. The degree of tissue excision necessary for sufficient sampling may result in additional reduction of testicular volume, especially in patients with pre-existing diminished testicular size due to underlying pathology [1]. Moreover, repeated TESE procedures may worsen testicular injury and further compromise endocrine function, leading to concerns regarding long-term consequences such as hypogonadism. Dasgupta et al. emphasize that managing hypogonadism in relation to fertility preservation necessitates meticulous attention to safety and follow-up, given that interventions may have enduring impacts on reproductive and hormonal health. A further limitation of TESE is the unpredictability of sperm retrieval, particularly in cases of idiopathic NOA, where the aetiology of spermatogenic failure is not well understood [35]. Despite the advantages, micro-TESE entails higher costs and limited availability, which restrict its accessibility compared with standard TESE. In order to reduce costs, the use of magnifying glasses instead of microscopes has proven to be at least as effective in several studies [36,37].

Overall, the predictive utility of AMH is constrained by inter-individual variability, overlapping values between successful and unsuccessful cases, and heterogeneity across studies [20,38,39]. Moreover, the observational design of most available studies limits the strength of the evidence. The Newcastle–Ottawa Scale indicated average quality, with small sample sizes and short follow-up as the main limitations. In addition, AMH values are not linearly related to spermatogenic status, and low levels do not universally preclude sperm retrieval.

Nevertheless, the negative predictive value of low serum AMH may help refine patient counseling. Tian et al. demonstrated that incorporating AMH into predictive models improved surgical decision-making for micro-TESE, guiding personalized consultations that considered both SRR and potential pregnancy outcomes [40]. While AMH emerged as a key predictor in their analysis, it should not be used as a standalone determinant. In women, for instance, very low AMH does not always preclude live birth, underscoring that biomarker thresholds must be interpreted within a broader clinical context [41,42]. Current evidence indicates that although AMH has emerged as a promising biomarker, its predictive value is not definitive and may be affected by various factors, including the underlying etiology of azoospermia, the presence of genetic abnormalities, and interactions with other Sertoli cell products such as inhibin B [1,11]. The observed discrepancy between AMH and inhibin B levels in cases of genetic and cytotoxic spermatogenic failure suggests that the reduction in AMH is not solely indicative of Sertoli cell quantity but may signify a distinct characteristic of specific types of testicular dysfunction. The creation of multifactorial predictive models represents a rational advancement, as highlighted by Kaltsas et al., who stress the importance of integrating molecular biomarkers with clinical and hormonal factors. These models may offer a more refined risk stratification for patients undergoing TESE, advancing beyond dependence on individual biomarkers [6]. The implementation of advanced diagnostics is currently constrained by cost and accessibility, especially in resource-limited environments. Recent studies indicate the potential value of dynamic or ratio-based biomarkers, such as the AMH/testosterone ratio, which may provide enhanced predictive performance compared to absolute AMH levels alone. Alfano et al. demonstrated that an elevated AMH/testosterone ratio may indicate a depletion of the germ cell reservoir, implying that composite indices could more effectively represent the complexities of testicular function in idiopathic non-obstructive azoospermia [12].

Studies examining combined biomarker approaches yielded conflicting results. Duvilla et al. demonstrated that while individual markers had low predictive value, a logistic regression model combining serum FSH, seminal inhibin B, and seminal AMH achieved a satisfying AUC of 0.985. This suggested potential synergy when integrating markers from different compartments [14]. In contrast, Mitchell et al. found that combining seminal AMH and inhibin B with serum FSH did not improve predictive value beyond individual markers [16]. Alfano et al. discovered that in serum-based methodologies, although multiple hormones were assessed (FSH, LH, testosterone, inhibin B, estradiol), the AMH/testosterone ratio yielded marginally superior predictive accuracy (95%) compared to AMH alone (93%) [12]. Both parameters maintained significance in multivariate analysis, with AMH exhibiting an odds ratio (OR) of 0.28 (95% confidence interval [CI]: 0.13–0.61) and AMH/tT displaying an OR of 0.00 (95% CI: 0–0.13). Decision curve analysis showed that both markers had a net clinical benefit, but AMH/tT had a small edge. Pozzi et al. conducted a multivariate analysis incorporating various variables (age, mean testicular volume, FSH, estradiol), revealing that lower AMH levels were independently correlated with positive SR after controlling for confounding factors (OR: 0.79; 95% CI: 0.64–0.93, *p* = 0.03) [5].

To our knowledge, the provided literature lacks comprehensive cost-effectiveness analyses; however, multiple studies indicate clinical net benefits and the potential to avert unnecessary surgeries, suggesting unquantified economic advantages. The research, which is available, on the other hand, supports using AMH as part of a multi-parameter decision process to cut down on unnecessary mTESEs and give better advice to patients [7,12]. Additionally, according to Zhang et al., the combination of inhibin B and AMH (INHB/AMH) enhanced predictive accuracy in one cohort and provided greater net benefit than either component individually [20]. The available literature does not contain formal clinical practice guideline statements endorsing or defining AMH use for TESE selection. AMH is best described in the current literature as an adjunctive biomarker to inform counseling and selection rather than a standalone guideline-mandated test.

Our analysis has several limitations. The main limitation of this meta-analysis arises from substantial heterogeneity across the included studies, as indicated by high I^2^ values, due to variability in patient populations, TESE techniques, and, importantly, the lack of standardization in AMH assay methods and measurement units. Additionally, the evidence base consists entirely of observational studies with moderate methodological quality, often hampered by small sample sizes and the absence of consistent AMH cutoff values. The overlapping AMH values between successful and unsuccessful TESE cases further limit the overall predictive usefulness of this single hormonal marker. As a result, these factors restrict the strength of the pooled evidence and the clinical usefulness of AMH as a standalone predictor. 

Taken together, our findings suggest that serum AMH reflects Sertoli cell function and provides valuable, though limited, information for predicting TESE outcomes in NOA. Its role should be considered complementary to other clinical and biochemical markers, rather than as a solitary predictor. Further large-scale, prospective studies with standardized assays are needed to establish clinically useful thresholds and to clarify its integration into predictive algorithms.

## 5. Conclusions

NOA remains a highly complex cause of male infertility, reflecting heterogeneous patterns of testicular dysfunction and spermatogenic failure. Our findings indicate that serum AMH is significantly associated with sperm retrieval outcomes, although its predictive value is limited by variability across studies and overlapping values between successful and unsuccessful cases. Novel molecular and proteomic markers, including microRNAs and extracellular vesicles, hold promise to complement hormonal assessments and improve predictive accuracy, but require validation in large, multicentre prospective studies. Standardization of assay protocols and establishment of clinically relevant thresholds are essential to enhance reproducibility and clinical applicability. Ultimately, integrating AMH with emerging biomarkers and clinical variables may support individualized counselling, reduce unnecessary invasive procedures, and improve reproductive success rates in men with NOA.

## Figures and Tables

**Table 1:** Succinct summary of the study features analysed in this research.

Author	Year	Patients (n)	Method of Sperm Retrieval	SRR (%)	NOS Score
Aboukhshaba et al. [11]	2021	46	mTESE	45.65	7
Alfano et al. [12]	2017	47	mTESE	48.93	7
Benderradji et al. [1]	2021	19	TESE	31.57	4
Deng et al. [13]	2023	168	mTESE	30.35	7
Duvilla et al. [14]	2008	26	TESE	52.3	7
Isikoglu et al. [15]	2006	24	TESE	54.00	7
Mitchell et al. [16]	2010	139	TESE	54.16	8
Mostafa et al. [17]	2007	40	TESE	52.50	8
Pozzi et al. [5]	2023	117	mTESE	48.71	8
Renault et al. [18]	2022	119	TESE	45.37	6
Xiong et al. [19]	2019	59	NA	76.27	4
Zhang et al. [20]	2023	502	mTESE	53.78	7

**Table 2:** Summary of studies that evaluated combinations of markers.

Study	Combined Markers	Model Type	Performance	Key Findings
Alfano et al. [12]	AMH/testosterone ratio	Logistic regression	AUC 95%	AMH/tT slightly better than AMH alone
Duvilla et al. [14]	Serum FSH + seminal Inhibin B + seminal AMH	Logistic regression	AUC 0.985	Combined markers are superior to individual models
Mitchell et al. [16]	Seminal AMH + seminal Inhibin B + serum FSH	Not specified	No improvement	The combination did not improve predictive value
Pozzi et al. [5]	AMH + age + testicular volume + FSH + estradiol	Not specified	AUC 70.3%	AMH remained significant after adjustment

## Data Availability

The data that support the findings of this study are available from the corresponding author upon reasonable request.

## Abbreviations

The following abbreviations are used in this manuscript:

AMH	Anti-Müllerian Hormone
TESE	testicular sperm extraction
NOA	non-obstructive azoospermia
FSH	follicle-stimulating hormone
LH	luteinizing hormone
ICSI	intracytoplasmic sperm injection
SRR	sperm retrieval rates
PRISMA	Preferred Reporting Items for Systematic Reviews and Meta-Analyses
PICO	Populations, Intervention, Comparison, and Outcome
